# Child eating behavior predicts body mass index after 1 year: results from the Swiss Preschooler’s Health Study (SPLASHY)

**DOI:** 10.3389/fpsyg.2024.1292939

**Published:** 2024-04-02

**Authors:** Yoan Mihov, Andrea H. Meyer, Tanja H. Kakebeeke, Kerstin Stülb, Amar Arhab, Annina E. Zysset, Claudia S. Leeger-Aschmann, Einat A. Schmutz, Susi Kriemler, Oskar G. Jenni, Jardena J. Puder, Nadine Messerli-Bürgy, Simone Munsch

**Affiliations:** ^1^Department of Psychology, Clinical Psychology and Psychotherapy, University of Fribourg, Freiburg, Switzerland; ^2^Department of Psychology, University of Basel, Basel, Switzerland; ^3^Child Development Center, University Children’s Hospital Zurich, Zurich, Switzerland; ^4^Children’s Research Center, University Children’s Hospital Zurich, Zurich, Switzerland; ^5^Obstetric Service, Department Woman-Mother-Child, Lausanne University Hospital, Lausanne, Switzerland; ^6^Epidemiology, Biostatistics and Prevention Institute, University of Zurich, Zurich, Switzerland; ^7^Institute of Psychology, FADO, University of Lausanne, Lausanne, Switzerland

**Keywords:** CEBQ, eating behavior, BMI, preschooler, SPLASHY

## Abstract

Child obesity is a growing global issue. Preventing early development of overweight and obesity requires identifying reliable risk factors for high body mass index (BMI) in children. Child eating behavior might be an important and malleable risk factor that can be reliably assessed with the parent-report Child Eating Behavior Questionnaire (CEBQ). Using a hierarchical dataset (children nested within child care centers) from a representative cohort of Swiss preschool children, we tested whether eating behavior, assessed with a 7-factor solution of the CEBQ, and BMI at baseline predicted the outcome BMI after 1 year, controlling for socioeconomic status (*n* = 555; 47% female; mean age = 3.9 years, range: 2.2–6.6; mean BMI = 16 kg/m^2^, range: 11.2–23; mean age- and sex-corrected *z*-transformed BMI, zBMI = 0.4, range −4 to +4.7). The statistical model explained 65.2% of zBMI at follow-up. Baseline zBMI was a strong positive predictor, uniquely explaining 48.8% of outcome variance. A linear combination of all CEBQ scales, taken together, explained 10.7% of outcome variance. Due to their intercorrelations, uniquely explained variance by any individual scale was of negligible clinical relevance. Only food responsiveness was a significant predictor, when accounting for all other predictors and covariates in the model, and uniquely explained only 0.4% of outcome variance. Altogether, our results confirm, extend, and refine previous research on eating behavior and zBMI in preschool children, by adjusting for covariates, accounting for intercorrelations between predictors, partitioning explained outcome variance, and providing standardized beta estimates. Our findings show the importance of carefully examining the contribution of predictors in multiple regression models for clinically relevant outcomes.

## 1 Introduction

According to the World Health Organization, the prevalence of obesity in children aged 5–19 years increased more than twice globally, from 2.9 to 6.8%, between 2016 and 2020 (WHO, 2021). This is a concerning trend as overweight and obesity are major risk factors for multiple health conditions imposing a high burden at the individual and societal levels (Afshin et al., 2017; Felisbino-Mendes et al., 2020; Boutari and Mantzoros, 2022).

Preventing early development of overweight and obesity requires identifying risk factors for high body mass index (BMI) in children. Child eating behavior appears to be a promising candidate, as it is established early in life and tends to remain stable (Powell et al., 2018). It can be reliably measured with the parent-report Child Eating Behavior Questionnaire (CEBQ), a 35-item assessment tool developed by Wardle et al. (2001), validated by Carnell and Wardle (2007), and adapted in various languages (Leuba et al., 2022). CEBQ assesses different aspects of eating behavior in eight scales: FR, food responsiveness (e.g., “Given the choice, my child would eat most of the time”); EF, enjoyment of food (e.g., “My child enjoys eating”); EOE, emotional overeating (e.g., “My child eats more when worried”); SR, satiety responsiveness (e.g., “My child gets full before his/her meal is finished”); SE, slowness in eating (e.g., “My child eats slowly”); EUE, emotional undereating (“My child eats less when s/he is upset”); FF, food fussiness (e.g., “My child refuses new foods at first”), and DD, desire to drink (“My child is always asking for a drink”). Conceptually, these aspects have been summarized into two dimensions ‘Food Approach’ (EF, EOE, DD and FR), that could lead to unhealthy overeating, overweight, and obesity, whereas ‘Food Avoidance’ (SR, SE, EUE and FF) could lead to undereating and underweight (Leuba et al., 2022).

A recent meta-analysis reported that child eating behavior, as assessed with the CEBQ, is related to BMI (Kininmonth et al., 2021). In cross-sectional studies, without adjustment for covariates, higher scores in the food approaching scales FR, EF, EOE, and DD corresponded to higher z-standardized BMI (zBMI), whereas higher scores in the food avoidant scales SR, SE, EUE, and FF corresponded to lower zBMI (Kininmonth et al., 2021; Omar et al., 2022). Unadjusted correlation estimates ranged from *r* = −0.21, for SR, to *r* = 0.22, for FR (Kininmonth et al., 2021). Whereas evidence from such cross-sectional studies consistently indicates that child eating behavior characteristics measured by the CEBQ and BMI share variance at a given timepoint, more longitudinal studies in representative samples are needed to show whether and how the child’s eating behavior can predict future BMI (Kininmonth et al., 2021). The results of Kininmonth and colleagues suggest that higher FR, EF, EOE, and DD are related to a higher future zBMI, whereas higher SR and SE predict a lower zBMI (Kininmonth et al., 2021). However, none of the longitudinal studies addressed the covariates BMI at baseline, socioeconomic status, and childcare center while also: (a) examining the unique variance explained by each CEBQ scale, as a measure of clinical significance, and (b) examining the unique variance explained by all CEBQ scales altogether, accounting for the intercorrelations of CEBQ scales. To address these issues, we investigated child eating behavior as a predictor of BMI with data from the nationally representative longitudinal Swiss Preschooler’s Health Study (SPLASHY), which assessed all CEBQ scales, BMI at baseline and one-year follow-up, thus allowing to control for socioeconomic status and childcare center.

Our hypothesis in this study was that baseline CEBQ scale scores and baseline BMI of the children predict the children’s BMI at one-year follow-up when controlling for socioeconomic status of the family. Rather than BMI, we chose zBMI, an age- and sex-corrected *z*-transformed BMI, to ensure comparability with other studies (Kininmonth et al., 2021). Because CEBQ scales assess different aspects of eating behaviors, we tested the effect of all scales individually as predictors in a multilevel linear model. To assess the practical relevance of our findings, we calculated uniquely explained variance by each CEBQ scale, as well as outcome variance explained by all CEBQ scales.

## 2 Methods

### 2.1 Study sample

SPLASHY is a multi-site prospective cohort study carried out in the French and German speaking regions of Switzerland (ISRCTN41045021; Messerli-Bürgy et al. (2016)). Participants were recruited through child care centers in five cantons: Aargau, Berne, Fribourg, Vaud, and Zurich, which allowed a recruitment of a nationally representative study sample. We aimed to recruit as representative a sample as possible with children from both rural and urban environments as well as from varying socioeconomic status (SES) strata. To this end, we selected cantons with a high population density that that jointly constituted about half of Swiss population in 2012. The sampling frame contained all childcare centers with children aged 5 years or less. We aimed at giving each childcare center the same sampling probability. The sampling procedure was not identical in each canton as cantons in Switzerland have different educational policies due to their high degree of political autonomy. For most cantons we sampled childcare centers with probability proportional to size (PPS), while, e.g., for the canton Vaud a stratified simple random sampling regime was used. To keep the effort and the benefit for childcare centers as balanced as possible, the 3-week tests were only carried out if a childcare center was able to provide a room to run testing on 3 weeks without daily routine being disturbed by the testing team. Furthermore, assessment was only carried out if deemed acceptable by the daycare center teams, as they were also asked to collect saliva samples from the participating children.

**Figure 1:**
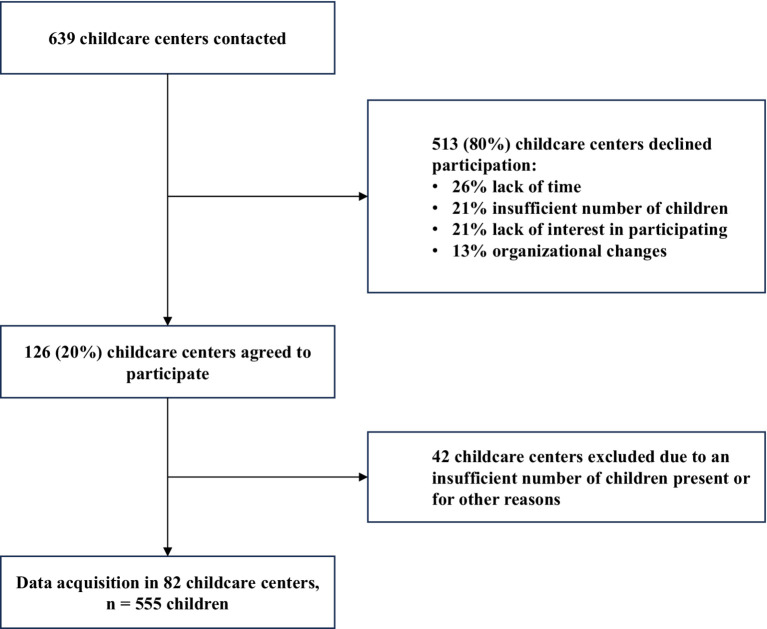
Flow chart of data acquisition.

The research team contacted a total of 639 childcare centers between January 2013 and October 2014, of which 20% agreed to participate (*n* = 126; Figure 1). Reasons to decline study participation for child care centers were: in 26% due to lack of time; in 21% due to an insufficient number of children, i.e., less than four, in the targeted age range present at the time; in 21% due to a lack of interest, and in 13% due to organizational changes. In addition, we excluded 42 centers after scheduling testing dates because of either an insufficient number of children, i.e., less than two, or for other reasons.

Data of 555 children were assessed in two data acquisition waves, at baseline (age 2–6 years) and at one-year follow-up. Children recruitment took place between November 2013 and October 2014 and was carried out in a final sample of 84 childcare centers across five Swiss cantons (Aargau, Bern, Fribourg, Vaud, Zurich).

SPLASHY was approved by all relevant local ethics committees (no 338/13 for the Ethical Committee of the Canton of Vaud as the main ethical committee) and the study was carried out in accordance with the Declaration of Helsinki. The parents of all participating children provided written informed consent before assessment.

Sample size calculations were based on the data simulation software MLPowSim 2 in combination with R, version 2.13.1, with 5,000 permutations per simulation. We thereby selected an effect size of *r* = 0.18 which was the minimum expected effect size to have a sufficiently high sample size to test our hypotheses. Further conditions were the statistical power 1–β = 0.8, α = 0.05, two-sided test. Based on a previous study (Bonvin et al., 2013) we presumed that around 12 children per child care center or 1,150 children in total would be present and could be invited on a given afternoon of testing. We assumed a participation rate of 40% or 500 children.

### 2.2 Assessment of child eating behavior

Eating behavior was assessed using the CEBQ, a 35-item instrument (Wardle et al., 2001; Carnell and Wardle, 2007). The German and French adaptation that we used displayed a 7-factor structure that was based on 30 items and comprised the following scales: food responsiveness, enjoyment of food, emotional overeating, satiety responsiveness, slowness in eating, emotional undereating, and food fussiness (Leuba et al., 2022). For this study, we calculated scale scores as the mean value of item scores in each scale according to the author’s guidelines (Carnell and Wardle, 2007). When calculating the mean, a maximum of one item response was allowed as a missing value. When two or more item responses from a single scale were missing the entire scale-score was declared as missing. Item responses represented five-stepped Likert scale-evaluations of statements about a child’s eating behavior.

### 2.3 Body mass index

The study team visited childcare centers at T1 and T2 to assess weight with a calibrated scale and height with a stadiometer. Although the BMI is calculated the same way for all individuals, normative BMI values vary with age and sex. Therefore, we chose the age- and sex-corrected z-transformed BMI measure as outcome variable, as proposed by the World Health Organization.^1^ BMI was assessed by the research team on the first day of measurements at the respective childcare center.

### 2.4 Socioeconomic status

Socioeconomic status was assessed as reported previously (Messerli-Bürgy et al., 2016; Kakebeeke et al., 2018; Zysset et al., 2018). For each child, the occupational status of each parent was rated according to the International Socio-Economic Index ISEI-08 (Ganzeboom, 2010). Ratings can vary between 16, indicating an unskilled worker, and 90, indicating a judge (Zysset et al., 2018). The higher SES of both parents was taken as a measure of the respective child’s SES.

### 2.5 Statistical analysis

We assessed the predictive value of baseline zBMI and CEBQ scores with a multilevel model. Multilevel models were required to account for the dependence in the data that may arise when children are assessed within childcare centers (i.e., data between children from the same child center are potentially more similar than those between two children from different childcare centers). Children thereby served as level-one and childcare centers as level-two units. The model contained the zBMI at follow-up as an outcome variable, zBMI at baseline and the seven CEBQ scale scores as predictors, and SES as a covariate (see Supplementary methods). Model fitting was carried out with the “lme” function (see Supplementary methods) as implemented in the package “nlme” (3.1.155) for R (all analyses were carried out in R, version 4.1.3; R Core Team, 2022). Partitioning of outcome variance explained by fixed effects and standardized beta weights were calculated with the “partR2” package, version 0.9.1.9000 (see Supplementary methods). Prior to model fitting, predictor multicollinearity was tested with the package “car” (3.0.12). Multicollinearity was considered acceptable because variance inflation factors of the seven predictors ranged between 1.03 and 2.22 and were thus far below the recommended threshold of 10.

The data were checked for potential outliers, using Cook’s distance measure. Critical values were defined according to the following formula: 4/(number of observations in the model − number of predictors − 1).

To deal with missing values we performed multiple data imputation according to van Buuren and Groothuis-Oudshoorn (2011) with the R-package “mice” (3.14.0). Multiple imputation has been repeatedly shown to lead to less biased results compared with, e.g., complete case analyses or analyses in which missing values are imputed using the last observation carried forward method (Schafer and Graham, 2002). We performed 100 imputations with a maximum of 20 iterations each, refitted our hierarchical model in each imputed dataset, and pooled the results from all refitted models. Imputation was carried out for all variables with missing values (*n* < 555) as reported in Table 1. In each imputation all missing values in all variables were imputed, resulting in a dataset without missing values for 555 participants. Model assumptions were checked by visually inspecting level-one and level-two residuals.

**Table 1:** Study sample characteristics.

Variable	Mean	SD	Min	Max	Valid cases
Age baseline	3.9	0.7	2.2	6.6	555
n female	262/555	–	–	–	555
BMI baseline	16.0	1.4	11.2	23.0	538
zBMI baseline	0.4	1.0	−4.0	4.7	538
BMI follow-up	15.8	1.4	11.6	25.8	363
zBMI follow-up	0.3	0.9	−3.3	5.4	362
SES	62.9	15.5	17.0	89.0	520
CEBQ-FR baseline	2.0	0.8	1.0	5.0	509
CEBQ-EO baseline	1.5	0.6	1.0	3.7	504
CEBQ-EF baseline	3.5	0.5	1.8	4.6	509
CEBQ-SR baseline	2.9	0.7	1.0	4.8	511
CEBQ-SE baseline	2.9	0.7	1.0	5.0	509
CEBQ-EU baseline	3.0	0.9	1.0	5.0	507
CEBQ-FF baseline	2.9	0.8	1.0	4.8	509

Mean, SD, Min and Max, the average, standard deviation minimal and maximal value, respectively; Valid Cases, the number of valid cases out of *n* = 555 participants; Age, the age in years; BMI, non-adjusted BMI values; zBMI, the age- and sex-adjusted *z*-transformed BMI at baseline and follow-up (see Methods); SES, the higher socioeconomic status among the child’s parents; CEBQ scales as follows: FR, food responsiveness; EO, emotional overeating; EF, enjoyment of food; SR, satiety responsiveness; SE, slowness in eating; EU, emotional undereating, FF, food fussiness.

## 3 Results

The total sample of 555 children comprised 262 (47%) girls and 293 boys. At the time of study enrollment children had an average age of 3.9 years (range: 2.2–6.6) and an average BMI of 16 kg/m^2^ (range: 11.2–23). The average zBMI was 0.4, ranging from −4 to 4.7 (Table 1; Supplementary Figure S1). The average SES-score was 62.9 (range: 17–89; Table 1), corresponding to a middle to high SES of the sample. Average CEBQ scale scores were in the range 1.53 (Emotional Overeating, Table 1; Supplementary Figure S2) – 3.52 (Enjoyment of Food, Table 1; Supplementary Figure S1), corresponding to average values for healthy children (Sleddens et al., 2008) (note, however, that in this study a 7-factor adaptation of the CEBQ was used). The intercorrelations between CEBQ scales at baseline and follow-up, as well as correlations between CEBQ and zBMI, are presented in Supplementary Figure S3.

Results after multiple imputation are summarized in Table 2 (*n* = 555, reported *p*-values not corrected for multiple comparisons). Higher zBMI at follow-up was associated with higher baseline zBMI (*p* < 0.001), and higher values of the CEBQ scale food responsiveness Scores (*p* = 0.01). For the remaining CEBQ scales the association with zBMI at follow-up was not significant (*p* > 0.05, all *p*-values uncorrected for multiple comparisons).

**Table 2:** Pooled results of model fitting after multiple imputation.

Variable	Estimate	Std. Error	*t*-value	DF	*p*-value
zBMI	0.720	0.035	20.751	194.010	<0.001
CEBQ-FR	0.137	0.053	2.589	228.028	0.010
CEBQ-EO	0.001	0.069	0.022	210.579	0.983
CEBQ-EF	−0.086	0.075	−1.146	235.530	0.253
CEBQ-SR	−0.070	0.055	−1.281	183.742	0.202
CEBQ-SE	−0.002	0.043	−0.055	212.277	0.956
CEBQ-EU	0.068	0.037	1.849	229.883	0.066
CEBQ-FF	−0.023	0.043	−0.536	211.640	0.593

zBMI, the age- and sex-adjusted *z*-transformed BMI at baseline (see Methods); CEBQ scales as follows: FR, food responsiveness; EO, emotional overeating; EF, enjoyment of food; SR, satiety responsiveness; SE, slowness in eating; EU, emotional undereating; FF, food fussiness. SES, the higher socioeconomic status among the child’s parents; Estimate and Std. Error, pooled model parameter estimates, after multiple imputation, and their corresponding standard errors; DF, degrees of freedom; *t*-values and *p*-values, each model parameter whereby *p*-values were not corrected for multiple comparisons; model fitted on *n* = 555 participants after data imputation.

We re-fitted the model in a subset comprising only cases without missing values (*n* = 323; Supplementary Table S1). This analysis corroborated the results yielded by multiple imputation: higher zBMI at follow-up was associated with higher baseline zBMI (*p* < 0.001; Supplementary Figure S4) and higher values of the CEBQ scale food responsiveness scores (*p* = 0.018; Supplementary Figure S5). None of the other six CEBQ scales was a significant predictor of zBMI at follow-up (all *p* > 0.05; Supplementary Table S1). The standardized regression coefficient was very high for zBMI at baseline, but small to very small for a linear combination of all CEBQ scales, including food responsiveness (see Supplementary methods, for details on calculation, and column “Beta weights” in Supplementary Table S1, for results). The model explained 65.2% of the total variance of the outcome, zBMI, at follow-up (marginal *R*^2^, i.e., variance explained by all fixed effects predictors and covariates in the model). The zBMI at baseline thereby uniquely explained 48.8% of the outcome variance, whereas the unique variance explained by individual CEBQ scales varied between 0 and 0.4%. Finally, the unique variance explained by all seven CEBQ scales together was 10.7%.

We identified 20 multivariate outliers in the subset without missing values, removed them and re-fitted our model, to further test the robustness of our findings (Supplementary Table S2). Again, higher zBMI at follow-up corresponded to higher baseline zBMI (*p* < 0.001) and higher values of the CEBQ scale food responsiveness (*p* = 0.004). In addition, higher zBMI at follow-up corresponded to lower baseline Satiety responsiveness scores (*p* = 0.032) and higher Enjoyment of Food scores (*p* = 0.024). For the remaining CEBQ scales the association with zBMI at follow-up was not significant (*p* > 0.05, all *p*-values uncorrected for multiple comparisons).

After model fitting, we applied false discovery rate-correction for multiple testing (Supplementary Table S5). We used the Benjamini-Yekutieli procedure because it does not assume test independence. In the pooled model, after data imputation (*n* = 555), baseline zBMI remained significant, whereas there was only a trend for statistical significance for baseline Food responsiveness (*p* = 0.087; Supplementary Table S5). When considering a smaller subset with only valid cases (*n* = 323), only the effect of baseline zBMI remained significant. In the subset of valid cases without multivariate outliers (*n* = 303) both baseline zBMI and Food responsiveness remained significant. Throughout, SES was a significant covariate.

We did not find evidence that participants who dropped out before follow-up differed from participants that completed study participation (Supplementary Tables S3, S4).

## 4 Discussion

In this study, we tested whether eating behavior, as measured with the CEBQ, and zBMI measured at baseline predict zBMI at one-year follow-up in preschool children. Our results corroborate and extend current research on the topic in four ways. First, we analyzed longitudinal, rather than cross-sectional, data from a representative cohort of Swiss preschool children. Second, when testing the influence of CEBQ-scales, our model accounted for the influence of the predictor baseline zBMI and the covariate SES while also considering the hierarchical nature of the data, i.e., that children were assessed within childcare centers. Third, we checked whether multivariate outliers or attrition bias might have influenced our results and found no evidence in support of this. Fourth, after carrying out statistical model fitting, we calculated the standardized beta weights for all predictors in the model. When considering the effect of the single scales of the CEBQ, higher baseline food responsiveness scores predict higher zBMI at one-year follow-up, but this effect was small and is of limited relevance for the clinical practice. Higher baseline zBMI was a statistically significant and clinically highly relevant predictor of zBMI at follow-up. FDR correction of all fitted models corroborated these conclusions. While baseline zBMI remained a significant predictor throughout, the effect of food responsiveness oscillated around significance threshold, being a trend in a large data sample after multiple imputation, non-significant in a smaller sample yielding a lower statistical power, and statistically significant after removing multivariate outliers from the smaller subset.

Food responsiveness is a measure of the tendency to eat in absence of physical hunger, which is likely to correspond to the biological and behavioral underpinnings of food related reward sensitivity and presents early in life (Stice et al., 2009). Within CEBQ, it is operationalized by the parents’ assessment of their child’s tendency to constantly ask for food and eat too much in general (Wardle et al., 2001). Several studies have investigated the predictive value of food responsiveness over different timespans, mostly in children aged 4–8 years, controlled for different covariates, and produced mixed findings (Kininmonth et al., 2021). In a sample of 35 2-year-old Australian children food responsiveness did not significantly predict BMI at age 4 years (Mallan et al., 2014). This study assessed maternal education but did not report on adjusting for this covariate when testing food responsiveness. In our study, we assessed SES and included it as a covariate. In a sample of 3,331 Dutch children, food responsiveness at age 4 years predicted BMI at age 10 years, when accounting for the covariates maternal educational level and household income (Derks et al., 2018). This relationship was no longer significant after adjusting for the covariate BMI at the age 4 years (Derks et al., 2018). Similarly, in our study, we found that when accounting for baseline zBMI, the unique variance explained by single CEBQ scales was low. In a sample of 418 British children aged 5–6 years there was a non-significant trend towards higher food responsiveness predicting higher BMI at age 6–8 years when accounting for the covariates age, sex, and birth weight (Parkinson et al., 2010). This study controlled for a two-stepped measure of economic status and for maternal educational status. The non-significant trend, after accounting for birth weight and economic status, is in line with our finding of low unique variance explained by food responsiveness. A Singaporean prospective study in younger children, with a sample of 210, showed that higher food responsiveness, measured with the baby version of the CEBQ (BEBQ), at 3 months-age predicted a higher zBMI between the ages of 6–15 months, but not 18 and 24 months, adjusted for the covariates maternal education and zBMI at birth (Quah et al., 2015). Food responsiveness, measured with the CEBQ, at 12 months did not predict BMI at 12–24 months (Quah et al., 2015). In a cohort of Greek children (*n* = 926) higher food responsiveness at age 4 years correlated with higher zBMI at age 6 years, but, based on a path analysis, the authors found no evidence for a significant unique predictive value of food responsiveness (Leventakou et al., 2022). This study used maternal education as a covariate (Leventakou et al., 2022). In contrast to food responsiveness, zBMI at age 4 years significantly predicted zBMI at age 6 years (Leventakou et al., 2022). This report is in line with our finding that zBMI is by far the strongest predictor of future zBMI. In another study, in 6-year-old Norwegian children (*n* = 675), higher food responsiveness predicted higher BMI increase over the following 2 years when controlling for parental BMI and SES (Steinsbekk and Wichstrøm, 2015). This study used a six-stepped measure of SES and a BMI standard deviation scores estimation procedure (Steinsbekk and Wichstrøm, 2015). A study of 113 low-income Hispanic children aged 4–5 years living in the United States examined the relationship between food responsiveness and BMI over three timepoints (Power et al., 2020). At timepoint 1 food responsiveness was not a significant predictor of zBMI 18 months later (timepoint 2) (Power et al., 2020). Interestingly, higher food responsiveness at timepoint 2 predicted higher BMI at an additional 16 or more months after this second assessment (Power et al., 2020).

One possible explanation for the inconclusive findings is that the predictive value of food responsiveness for BMI might increase with age. Of the above-mentioned studies, two carried out in children below the age of three found no significant relationship (Mallan et al., 2014; Quah et al., 2015), whereas three in the age group 5–6 years reported significant or a trend-to-significant relationship (Parkinson et al., 2010; Steinsbekk and Wichstrøm, 2015; Power et al., 2020). Studies in the age range in-between, including our own investigation, reported either a non-significant positive relationship or a positive relationship that was strongly diminished or absent when correcting for covariates such as baseline BMI (Derks et al., 2018; Power et al., 2020; Leventakou et al., 2022). Overall, this might suggest a transition in the predictive value of food responsiveness in the age range 4–6.

What might increase the predictive value of food responsiveness for BMI during the transition to primary school? According to the behavioral susceptibility theory (BST), the heritability of BMI is mediated by a phenotype characterized by appetite, sensitivity to satiety, and the resulting eating behavior (Llewellyn and Wardle, 2015; Llewellyn and Fildes, 2017). A growing body of evidence suggests that various neurobiological systems, most notably dopamine and glutamate, are involved in the regulation of appetite and eating, and may mediate the heritability of obesity (Smith and Robbins, 2013; Mihov and Hasler, 2016; Yohn et al., 2019). Importantly, these neurotransmitter systems dynamically respond to environmental changes, in accordance with the gene–environment interaction posed by BST (Llewellyn and Wardle, 2015). In preclinical rodent models, limited access to food, shock stress, and psychosocial stress induce hyperphagia and body weight gain (Razzoli et al., 2017). These experimental environmental effects are mediated, at least in part, by glutamate signaling (Oliveira et al., 2021). Preclinical evidence is consistent with altered glutamate signaling found in bulimia nervosa (Mihov et al., 2020). Thus, stressful environmental changes might drive, or exacerbate pre-existing, changes in dopamine and glutamate signaling that contribute to dysregulated eating and weight gain. Exposure to environmental stress and its impacts, in turn, may vary greatly with a group of interrelated factors, such as low SES, low maternal education, and high food insecurity. In our study, lower SES was associated with higher zBMI. Recently, distinct eating temperaments were suggested, among which “avid eating” (reflecting high food approach) was associated with higher food responsiveness and higher food insecurity (Pickard et al., 2023). Altogether, lower SES might increase exposure to stressful events, especially during transition phases such as to primary school, combine with genetically mediated pre-existing vulnerabilities and unlock a pattern of neural and behavioral changes leading to overeating and weight gain, in accordance with the BST (Llewellyn and Wardle, 2015; Llewellyn and Fildes, 2017).

In a subsample of participants without any missing values in any of the model variables and after exclusion of multivariate outliers, in addition to higher baseline zBMI and higher food responsiveness, lower satiety response and higher emotional undereating were significant predictors of future zBMI. In their meta-analysis, Kininmonth et al. (2021) found, for prospective studies, a positive relationship between satiety response and future zBMI but no significant relationship between emotional undereating and future zBMI. Since these findings emerged only after several steps of subsampling, were not supported by multiple imputation estimation, nor were of clinical relevance, their interpretation and discussion warrant caution.

In addition to the dichotomous question of whether food responsiveness is a “significant” or “non-significant” predictor of future BMI, we quantified the future outcome variance explained by food responsiveness. Our findings show that food responsiveness is a statistically significant predictor of future BMI but fails to explain enough variance to be of clinical relevance when accounting for the influence of baseline zBMI and all other CEBQ scales. Due to the partly considerable intercorrelations among different CEBQ scales, uniquely explained outcome variance was low for each individual CEBQ scale, when regarded alone. In contrast, a linear combination of all CEBQ scales explained nearly 11 percent of follow-up zBMI and can be considered as a clinically meaningful predictor. This finding supports multivariate attempts to approach eating behavior, as temperamental traits (Pickard et al., 2023), or a “composite obesogenic appetite score” (Renier et al., 2024).

Several aspects of our study can be regarded as limitations. First, we calculated CEBQ scores using a 7-factor adaptation of the original scale (Leuba et al., 2022). This specific factor solution limits direct comparisons to studies that used the original 8-scale 35-items CEBQ (Wardle et al., 2001; Carnell and Wardle, 2007). However, the fact that food responsiveness was a significant positive predictor of future BMI supports the validity of our factor solution. Second, data loss due to dropouts could have influenced results. We addressed this issue with a multiple imputation analysis, an analysis of a subsample without any missing value in the model variables, and a subset of the latter sample, without multivariate outliers. All three analyses converged into showing a predictive value of food responsiveness and arguing against attrition-caused influence on findings.

Ethnic and cross-cultural differences can translate to differences in parents’ views on, ratings of, and reaction to child eating behavior, and these relations can be partly mediated by co-occurring socioeconomic differences (Kumanyika, 2008; Musher-Eizenman et al., 2009; Blissett and Bennett, 2013; Niemeier et al., 2017; Somaraki et al., 2017; Yılmaz et al., 2019; Rohit et al., 2021). Therefore, ethnically and culturally highly homogeneous study samples might increase statistical power for effects specific to one ethnicity or culture while, at the same time, limiting generalizability of findings to different ethnicities and cultures. Our sample, recruited in cantons that constitute approximately one half of Swiss population, can be regarded as representative of Switzerland (Messerli-Bürgy et al., 2016). Since Switzerland is culturally and linguistically diverse, we assume our sample reflected this diversity, although we did not assess ethnicity and cultural background to confirm this. Cultural and linguistic diversity inherent so Switzerland implies generalizability of our findings to surrounding states while suggesting limited generalizability to more distinct cultures, and possibly to societies with greater socioeconomic disparities. More generally, this might reflect an unavoidable trade-off in cohort studies between specificity and generalizability which is to be considered when designing recruitment procedures.

Two methodological issues likely influence the results of all longitudinal studies on CEBQ and zBMI in preschool children, ours included: the specific parent providing eating behavior assessment (i.e., mother or father) and the time between baseline CEBQ assessment and follow-up zBMI measurement. Regarding the first issue, it should be noted that fathers are rarely interviewed (Vollmer et al., 2015). In our sample, only 14% of CEBQ assessments were provided by fathers. This is an important research gap, as fathers too can influence child eating behavior and not interviewing them might fail to account for an important factor (Litchford et al., 2020). The precise differences between the contributions of mothers and fathers to eating behavior and BMI development appear complex and warrant further research (Wake et al., 2007; Berge et al., 2010; Hoffman et al., 2014). In addition, further studies are needed to investigate whether mothers and fathers base their assessment on different implicit norms and provide different assessments of the same child eating behavior with the CEBQ (De-Jongh González et al., 2021). The second general methodological issue refers to how the influence of eating behavior on BMI changes over the course of life. If eating behavior is a trait manifesting early in life, its contribution to BMI may grow over time, as children gain independence and start to self-determine their eating behavior (Llewellyn et al., 2010). While our study focused on two measurements with a one-year follow-up in preschool children, longitudinal investigations over multiple timepoints and an extended period of the child’s development are needed to address this point.

While the validity of body mass and height measures appears unobjectionable, especially when compared to subjective reports on behavior, a case has been made that the use of non-calibrated instruments can introduce error and overestimate the variance of anthropometric measures in population-based investigations (Biehl et al., 2013). This issue was directly addressed by careful investigations demonstrating acceptable reliability for height measurements with a portable stadiometer (McKenna et al., 2013; Baharudin et al., 2017). Based on these findings and the fact that we used a calibrated scale and a stadiometer, the methods we applied for anthropometric measurements can be regarded as adequate.

In conclusion, we found that in Swiss preschool children aged 2–6 years higher zBMI and higher food responsiveness predicted higher zBMI after 1 year, when accounting for socioeconomic status. Baseline zBMI was by far the most important predictor. A linear combination of all CEBQ scales explained a notable fraction of follow-up zBMI variance and can hence be considered clinically meaningful when predicting BMI in children. These results underline the importance of considering covariates, intercorrelation patterns and partitioning explained variance when fitting multiple regression models to predict clinically relevant outcomes.

## Data availability statement

Data supporting the conclusions of this article will be made available upon request.

## Ethics statement

The studies involving humans were approved by no 338/13 for the Ethical Committee of the Canton of Vaud as the main ethical committee. The studies were conducted in accordance with the local legislation and institutional requirements. Written informed consent for participation in this study was provided by the participants’ legal guardians/next of kin.

## Author contributions

YM: Writing – original draft, Writing – review & editing. AM: Writing – original draft, Writing – review & editing. TK: Writing – review & editing. KS: Writing – review & editing. AA: Writing – review & editing. AZ: Writing – review & editing. CL-A: Writing – review & editing. ES: Writing – review & editing. SK: Supervision, Writing – review & editing. OJ: Supervision, Writing – review & editing. JP: Supervision, Writing – review & editing. NM-B: Supervision, Writing – review & editing. SM: Supervision, Writing – original draft, Writing – review & editing.

## Footnotes

^1^ https://www.who.int/tools/child-growth-standards

## References

[ref1] Afshin A. Forouzanfar M. H. Reitsma M. B. Sur P. Estep K. Lee A.. (2017). Health effects of overweight and obesity in 195 countries over 25 years. N. Engl. J. Med. 377, 13–27. doi: 10.1056/NEJMoa1614362, PMID: 28604169 PMC5477817

[ref2] Baharudin A. Ahmad M. H. Naidu B. M. Hamzah N. R. Zaki N. A. M. Zainuddin A. A.. (2017). Reliability, technical error of measurement and validity of height measurement using portable stadiometer. Pertanika J. Sci. Technol. 25, 675–686.

[ref3] Berge J. M. Wall M. Loth K. Neumark-Sztainer D. (2010). Parenting style as a predictor of adolescent weight and weight-related behaviors. J. Adolesc. Health 46, 331–338. doi: 10.1016/j.jadohealth.2009.08.004, PMID: 20307821 PMC2844861

[ref4] Biehl A. Hovengen R. Meyer H. E. Hjelmesaeth J. Meisfjord J. Grøholt E. K.. (2013). Impact of instrument error on the estimated prevalence of overweight and obesity in population-based surveys. BMC Public Health 13:146. doi: 10.1186/1471-2458-13-146, PMID: 23413839 PMC3606378

[ref5] Blissett J. Bennett C. (2013). Cultural differences in parental feeding practices and children's eating behaviours and their relationships with child BMI: a comparison of black afro-Caribbean, white British and white German samples. Eur. J. Clin. Nutr. 67, 180–184. doi: 10.1038/ejcn.2012.198, PMID: 23232584

[ref6] Bonvin A. Barral J. Kakebeeke T. H. Kriemler S. Longchamp A. Schindler C.. (2013). Effect of a governmentally-led physical activity program on motor skills in young children attending child care centers: a cluster randomized controlled trial. Int. J. Behav. Nutr. Phys. Act. 10:90. doi: 10.1186/1479-5868-10-90, PMID: 23835207 PMC3724593

[ref7] Boutari C. Mantzoros C. S. (2022). A 2022 update on the epidemiology of obesity and a call to action: as its twin COVID-19 pandemic appears to be receding, the obesity and dysmetabolism pandemic continues to rage on. Metabolism 133:155217. doi: 10.1016/j.metabol.2022.155217, PMID: 35584732 PMC9107388

[ref8] Carnell S. Wardle J. (2007). Measuring behavioural susceptibility to obesity: validation of the child eating behaviour questionnaire. Appetite 48, 104–113. doi: 10.1016/j.appet.2006.07.075, PMID: 16962207

[ref9] De-Jongh González O. Tugault-Lafleur C. N. O'Connor T. M. Hughes S. O. Mâsse L. C. (2021). Are fathers' and mothers' food parenting practices differentially associated with children's eating behaviors? Appetite 166:105434. doi: 10.1016/j.appet.2021.105434, PMID: 34107293

[ref10] Derks I. P. M. Sijbrands E. J. G. Wake M. Qureshi F. van der Ende J. Hillegers M. H. J.. (2018). Eating behavior and body composition across childhood: a prospective cohort study. Int. J. Behav. Nutr. Phys. Act. 15:96. doi: 10.1186/s12966-018-0725-x, PMID: 30285789 PMC6167809

[ref11] Felisbino-Mendes M. S. Cousin E. Malta D. C. Machado Í. E. Ribeiro A. L. P. Duncan B. B.. (2020). The burden of non-communicable diseases attributable to high BMI in Brazil, 1990-2017: findings from the global burden of disease study. Popul. Health Metrics 18:18. doi: 10.1186/s12963-020-00219-y, PMID: 32993699 PMC7525961

[ref12] Ganzeboom H. B. (2010). A New International Socio-Economic Index (ISEI) of Occupational Status for the International Standard Classification of Occupation 2008 (ISCO-08). Constructed with Data from the ISSP 2002–2007. Annual Conference of International Social Survey Programme, Lisbon.

[ref13] Hoffman E. R. Bentley M. E. Hamer R. M. Hodges E. A. Ward D. S. Bulik C. M. (2014). A comparison of infant and toddler feeding practices of mothers with and without histories of eating disorders. Matern. Child Nutr. 10, 360–372. doi: 10.1111/j.1740-8709.2012.00429.x, PMID: 22784046 PMC3473145

[ref14] Kakebeeke T. H. Zysset A. E. Messerli-Bürgy N. Chaouch A. Stülb K. Leeger-Aschmann C. S.. (2018). Impact of age, sex, socioeconomic status, and physical activity on associated movements and motor speed in preschool children. J. Clin. Exp. Neuropsychol. 40, 95–106. doi: 10.1080/13803395.2017.1321107, PMID: 28548032

[ref15] Kininmonth A. Smith A. Carnell S. Steinsbekk S. Fildes A. Llewellyn C. (2021). The association between childhood adiposity and appetite assessed using the child eating behavior questionnaire and baby eating behavior questionnaire: a systematic review and meta-analysis. Obes. Rev. 22:e13169. doi: 10.1111/obr.13169, PMID: 33554425

[ref16] Kumanyika S. K. (2008). Environmental influences on childhood obesity: ethnic and cultural influences in context. Physiol. Behav. 94, 61–70. doi: 10.1016/j.physbeh.2007.11.019, PMID: 18158165

[ref17] Leuba A. L. Meyer A. H. Kakebeeke T. H. Stülb K. Arhab A. Zysset A. E.. (2022). The relationship of parenting style and eating behavior in preschool children. BMC Psychol 10:275. doi: 10.1186/s40359-022-00981-8, PMID: 36419113 PMC9682652

[ref18] Leventakou V. Herle M. Kampouri M. Margetaki K. Vafeiadi M. Kogevinas M.. (2022). The longitudinal association of eating behaviour and ADHD symptoms in school age children: a follow-up study in the RHEA cohort. Eur. Child Adolesc. Psychiatry 31, 511–517. doi: 10.1007/s00787-021-01720-x, PMID: 33599859 PMC8634555

[ref19] Litchford A. Savoie Roskos M. R. Wengreen H. (2020). Influence of fathers on the feeding practices and behaviors of children: a systematic review. Appetite 147:104558. doi: 10.1016/j.appet.2019.104558, PMID: 31870933

[ref20] Llewellyn C. H. Fildes A. (2017). Behavioural susceptibility theory: professor Jane Wardle and the role of appetite in genetic risk of obesity. Curr. Obes. Rep. 6, 38–45. doi: 10.1007/s13679-017-0247-x, PMID: 28236287 PMC5359365

[ref21] Llewellyn C. H. van Jaarsveld C. H. Johnson L. Carnell S. Wardle J. (2010). Nature and nurture in infant appetite: analysis of the Gemini twin birth cohort. Am. J. Clin. Nutr. 91, 1172–1179. doi: 10.3945/ajcn.2009.28868, PMID: 20335548

[ref22] Llewellyn C. Wardle J. (2015). Behavioral susceptibility to obesity: gene-environment interplay in the development of weight. Physiol. Behav. 152, 494–501. doi: 10.1016/j.physbeh.2015.07.006, PMID: 26166156

[ref23] Mallan K. M. Nambiar S. Magarey A. M. Daniels L. A. (2014). Satiety responsiveness in toddlerhood predicts energy intake and weight status at four years of age. Appetite 74, 79–85. doi: 10.1016/j.appet.2013.12.001, PMID: 24316574

[ref24] McKenna L. Straker L. Smith A. (2013). The inter-tester reliability of anthropometric measurement with portable tools. Eur. J. Phys. 15, 34–41. doi: 10.3109/14038196.2012.752522

[ref25] Messerli-Bürgy N. Kakebeeke T. H. Arhab A. Stülb K. Zysset A. E. Leeger-Aschmann C. S.. (2016). The Swiss preschoolers’ health study (SPLASHY): objectives and design of a prospective multi-site cohort study assessing psychological and physiological health in young children. BMC Pediatr. 16:85. doi: 10.1186/s12887-016-0617-7 27390933 PMC4939002

[ref26] Mihov Y. Hasler G. (2016). Negative allosteric modulators of metabotropic glutamate receptors subtype 5 in addiction: a therapeutic window. Int. J. Neuropsychopharmacol. 19:pyw002. doi: 10.1093/ijnp/pyw002, PMID: 26802568 PMC4966271

[ref27] Mihov Y. Treyer V. Akkus F. Toman E. Milos G. Ametamey S. M.. (2020). Metabotropic glutamate receptor 5 in bulimia nervosa. Sci. Rep. 10:6374. doi: 10.1038/s41598-020-63389-7, PMID: 32286451 PMC7156702

[ref28] Musher-Eizenman D. R. de Lauzon-Guillain B. Holub S. C. Leporc E. Charles M. A. (2009). Child and parent characteristics related to parental feeding practices. A cross-cultural examination in the US and France. Appetite 52, 89–95. doi: 10.1016/j.appet.2008.08.007, PMID: 18789986 PMC3808174

[ref29] Niemeier B. S. Duan Y. P. Shang B. R. Yang J. (2017). Parental influences on weight-related health behaviors in western and eastern cultures. Child Care Health Dev. 43, 259–266. doi: 10.1111/cch.12438 28074491

[ref30] Oliveira T. P. D. Gonçalves B. D. C. Oliveira B. S. de Oliveira A. C. P. Reis H. J. Ferreira C. N.. (2021). Negative modulation of the metabotropic glutamate receptor type 5 as a potential therapeutic strategy in obesity and binge-like eating behavior [original research]. Front. Neurosci. 15:631311. doi: 10.3389/fnins.2021.631311, PMID: 33642987 PMC7902877

[ref31] Omar O. M. Massoud M. N. Ibrahim A. G. Khalaf N. A. (2022). Effect of early feeding practices and eating behaviors on body composition in primary school children. World J. Pediatr. 18, 613–623. doi: 10.1007/s12519-022-00559-9, PMID: 35666456 PMC9169027

[ref32] Parkinson K. N. Drewett R. F. Le Couteur A. S. Adamson A. J. (2010). Do maternal ratings of appetite in infants predict later child eating behaviour questionnaire scores and body mass index? Appetite 54, 186–190. doi: 10.1016/j.appet.2009.10.007, PMID: 19887093

[ref33] Pickard A. Croker H. Edwards K. Farrow C. Haycraft E. Herle M.. (2023). Identifying an avid eating profile in childhood: associations with temperament, feeding practices and food insecurity. Appetite 191:107050. doi: 10.1016/j.appet.2023.107050, PMID: 37793473

[ref34] Powell F. Farrow C. Meyer C. Haycraft E. (2018). The stability and continuity of maternally reported and observed child eating behaviours and feeding practices across early childhood. Int. J. Environ. Res. Public Health 15:1017. doi: 10.3390/ijerph15051017, PMID: 29783638 PMC5982056

[ref35] Power T. G. Hidalgo-Mendez J. Fisher J. O. O'Connor T. M. Micheli N. Hughes S. O. (2020). Obesity risk in Hispanic children: bidirectional associations between child eating behavior and child weight status over time. Eat. Behav. 36:101366. doi: 10.1016/j.eatbeh.2020.101366, PMID: 31962209 PMC7044049

[ref36] Quah P. L. Chan Y. H. Aris I. M. Pang W. W. Toh J. Y. Tint M. T.. (2015). Prospective associations of appetitive traits at 3 and 12 months of age with body mass index and weight gain in the first 2 years of life. BMC Pediatr. 15:153. doi: 10.1186/s12887-015-0467-8, PMID: 26459321 PMC4603814

[ref37] R Core Team. (2022). R: A Language and Environment for Statistical Computing. R Foundation for Statistical Computing, Vienna, Austria.

[ref38] Razzoli M. Pearson C. Crow S. Bartolomucci A. (2017). Stress, overeating, and obesity: insights from human studies and preclinical models. Neurosci. Biobehav. Rev. 76, 154–162. doi: 10.1016/j.neubiorev.2017.01.026, PMID: 28292531 PMC5403578

[ref39] Renier T. J. Yeum D. Emond J. A. Lansigan R. K. Ballarino G. A. Carlson D. D.. (2024). Elucidating pathways to pediatric obesity: a study evaluating obesity polygenic risk scores related to appetitive traits in children. Int. J. Obes. 48, 71–77. doi: 10.1038/s41366-023-01385-3, PMID: 37736781 PMC10841756

[ref40] Rohit A. Kirkham R. McCarthy L. Puruntatameri V. Maple-Brown L. Brimblecombe J. (2021). Exploring differences in perceptions of child feeding practices between parents and health care professionals: a qualitative study. BMC Public Health 21:1449. doi: 10.1186/s12889-021-11493-2, PMID: 34301222 PMC8299622

[ref41] Schafer J. L. Graham J. W. (2002). Missing data: our view of the state of the art. Psychol. Methods 7, 147–177. doi: 10.1037/1082-989X.7.2.147 12090408

[ref42] Sleddens E. F. Kremers S. P. Thijs C. (2008). The children's eating behaviour questionnaire: factorial validity and association with body mass index in Dutch children aged 6-7. Int. J. Behav. Nutr. Phys. Act. 5:49. doi: 10.1186/1479-5868-5-49, PMID: 18937832 PMC2612017

[ref43] Smith D. G. Robbins T. W. (2013). The neurobiological underpinnings of obesity and binge eating: a rationale for adopting the food addiction model. Biol. Psychiatry 73, 804–810. doi: 10.1016/j.biopsych.2012.08.026, PMID: 23098895

[ref44] Somaraki M. Eli K. Ek A. Lindberg L. Nyman J. Marcus C.. (2017). Controlling feeding practices and maternal migrant background: an analysis of a multicultural sample. Public Health Nutr. 20, 848–858. doi: 10.1017/S1368980016002834, PMID: 27866503 PMC10261564

[ref45] Steinsbekk S. Wichstrøm L. (2015). Predictors of change in BMI from the age of 4 to 8. J. Pediatr. Psychol. 40, 1056–1064. doi: 10.1093/jpepsy/jsv052, PMID: 26050242

[ref46] Stice E. Spoor S. Ng J. Zald D. H. (2009). Relation of obesity to consummatory and anticipatory food reward. Physiol. Behav. 97, 551–560. doi: 10.1016/j.physbeh.2009.03.020, PMID: 19328819 PMC2734415

[ref47] van Buuren S. Groothuis-Oudshoorn K. (2011). Mice: multivariate imputation by chained equations in R. J. Stat. Softw. 45, 1–67. doi: 10.18637/jss.v045.i03

[ref48] Vollmer R. L. Adamsons K. Foster J. S. Mobley A. R. (2015). Association of fathers' feeding practices and feeding style on preschool age children's diet quality, eating behavior and body mass index. Appetite 89, 274–281. doi: 10.1016/j.appet.2015.02.021, PMID: 25700629

[ref49] Wake M. Nicholson J. M. Hardy P. Smith K. (2007). Preschooler obesity and parenting styles of mothers and fathers: Australian national population study. Pediatrics 120, e1520–e1527. doi: 10.1542/peds.2006-3707, PMID: 18055667

[ref50] Wardle J. Guthrie C. A. Sanderson S. Rapoport L. (2001). Development of the Children's eating behaviour questionnaire. J. Child Psychol. Psychiatry 42, 963–970. doi: 10.1111/1469-7610.00792 11693591

[ref51] WHO. (2021). Call for Experts: WHO Guideline Development Group on Treatment of Children and Adolescents with Obesity. Geneva, Switzerland: World Health Organization.

[ref52] Yılmaz N. G. Renders C. M. Nicolaou M. Vrijkotte T. G. M. (2019). The explanatory role of maternal feeding practices: do they explain ethnic differences in body weight of preadolescents? Appetite 142:104354. doi: 10.1016/j.appet.2019.104354, PMID: 31295505

[ref53] Yohn S. E. Galbraith J. Calipari E. S. Conn P. J. (2019). Shared behavioral and Neurocircuitry disruptions in drug addiction, obesity, and binge eating disorder: focus on group I mGluRs in the mesolimbic dopamine pathway. ACS Chem. Neurosci. 10, 2125–2143. doi: 10.1021/acschemneuro.8b00601, PMID: 30933466 PMC7898461

[ref54] Zysset A. E. Kakebeeke T. H. Messerli-Bürgy N. Meyer A. H. Stülb K. Leeger-Aschmann C. S.. (2018). Predictors of executive functions in preschoolers: findings from the SPLASHY study. Front. Psychol. 9:2060. doi: 10.3389/fpsyg.2018.02060, PMID: 30420823 PMC6216414

