# Supplementary material for: Child eating behavior predicts body mass index after 1 year: results from the Swiss Preschooler’s Health Study (SPLASHY)
Source: Front Psychol. 2024 Apr 2;15:1292939. doi: 10.3389/fpsyg.2024.1292939 (PMC11019003; doi:10.3389/fpsyg.2024.1292939)
Supplement: Supplementary file 1 [file Data_Sheet_1.PDF]

Supplementary Material

1 Supplementary Methods

Hierarchical model formulation

We tested our hypothesis with the following model formulation:

```
mod.01 <- nlme::lme(zBMI.at.followup ~  zBMI.at.baseline +
           a_CEBQ.fr + a_CEBQ.eo +
           a_CEBQ.ef + a_CEBQ.sr +
           a_CEBQ.se + a_CEBQ.eu +
           a_CEBQ.ff + SES,
          random =~ 1 | childcare.center,
           data=splashy.data,
           na.action="na.exclude")
```

Calculation of Standardized Beta Weights

We computed standardized beta weights in two steps. First, we refitted the model using the R-package lme4 in a sample with no missing values in any of the model variables (n = 323):

```
modBM <- lmer(zBMI.at.followup ~  zBMI.at.baseline +
                        a_CEBQ.fr + a_CEBQ.eo +
                        a_CEBQ.ef + a_CEBQ.sr +
                        a_CEBQ.se + a_CEBQ.eu +
                        a_CEBQ.ff + SES +
                       (1|childcare.center),
                       data=splashy.data,
                       na.action="na.exclude")
```

In a second step, we calculated standardized beta weights as follows:

```
R2_BM.20 <- partR2(modBM,
                   partvars = c("a_CEBQ.fr",
                                "a_CEBQ.eo",
                                "a_CEBQ.ef",
                                "a_CEBQ.sr",
                                "a_CEBQ.se",
                                "a_CEBQ.eu",
                                "a_CEBQ.ff",
                                "a_WHOzscore",
                                "a_isei_max"
                   ),
                   R2_type = "marginal",
                   nboot = 20,
                   max_level=2
)
```

2 Supplementary Tables

Supplementary Table 1. Model Fitted in a Subset Without Missing Values

Variable	Estimate	Std.Error	Beta Weights	DF	t-value	p-value
**zBMI**	0.745	0.035	0.75	232	21.225	< 0.001
**CEBQ-FR**	0.130	0.055	0.11	232	2.373	0.018
**CEBQ-EO**	0.010	0.070	0.01	232	0.148	0.883
**CEBQ-EF**	-0.100	0.076	-0.05	232	-1.308	0.192
**CEBQ-SR**	-0.073	0.052	-0.05	232	-1.410	0.160
**CEBQ-SE**	0.011	0.042	0.01	232	0.270	0.787
**CEBQ-EU**	0.067	0.036	0.07	232	1.839	0.067
**CEBQ-FF**	-0.029	0.042	-0.03	232	-0.684	0.495

zBMI refers to the age- and sex-adjusted z-transformed BMI at baseline (see Methods); ***CEBQ scales*** as follows: ***FR*** refers to Food responsiveness, ***EO*** refers to Emotional overeating, ***EF*** refers to Enjoyment of food, ***SR*** refers to Satiety responsiveness, ***SE*** refers to Slowness in eating, ***EU*** refers to Emotional undereating, and ***FF*** refers to Food fussiness; ***Estimate*** and ***Std.Error*** refer to model parameter estimates and their corresponding standard errors, as calculated with the package nlme; ***Beta Weights*** refer to standardized beta weights, as calculated with the package partR2; ***DF*** refers to degrees of freedom, ***t-values*** and ***p-values*** refer to each model parameter whereby p-values were not corrected for multiple comparisons; ***p-values*** were not corrected for multiple comparisons; model fitted on ***n = 323*** participants (no missing values).

Supplementary Table 2. Model Fitted in a Subset Without Missing Values After Exclusion of Outliers

Variable	Estimate	Std.Error	DF	t-value	p-value
**adjBMI**	0.781	0.031	212	25.550	< 0.001
**CEBQ-FR**	0.126	0.043	212	2.896	0.004
**CEBQ-EO**	-0.064	0.056	212	-1.142	0.255
**CEBQ-EF**	-0.085	0.060	212	-1.406	0.161
**CEBQ-SR**	-0.087	0.040	212	-2.153	0.032
**CEBQ-SE**	-0.004	0.032	212	-0.129	0.897
**CEBQ-EU**	0.064	0.028	212	2.278	0.024
**CEBQ-FF**	-0.022	0.033	212	-0.667	0.505

zBMI refers to the age- and sex-adjusted z-transformed BMI at baseline (see Methods); ***CEBQ scales*** as follows: ***FR*** refers to Food responsiveness, ***EO*** refers to Emotional overeating, ***EF*** refers to Enjoyment of food, ***SR*** refers to Satiety responsiveness, ***SE*** refers to Slowness in eating, ***EU*** refers to Emotional undereating, and ***FF*** refers to Food fussiness; ***Estimate*** and ***Std.Error*** refer to model parameter estimates and their corresponding standard errors; ***DF*** refers to degrees of freedom, ***t-values*** and ***p-values*** refer to each model parameter whereby p-values were not corrected for multiple comparisons; ***p-values*** were not corrected for multiple comparisons; model fitted on ***n = 303*** participants (no missing values).

Supplementary Table 3: Descriptive statistics of participants with complete data (n = 323) vs. participants with data containing at least one missing value in any of the model variables.

Variable	Mean.C	SD.C	Min.C	Max.C	Val.C	Mean.N	SD.N	Min.N	Max.N	Val.N	df	statistic	p-val
**Age baseline**	3.8	0.7	2.5	6.6	323	3.9	0.7	2.2	6.2	232	480.742	-0.785	0.433
**n Female**	148	-	-	-	323	114	-	-	-	232	1	0.471	0.493
**BMI baseline**	16.0	1.3	12.3	22.7	323	16.1	1.4	11.2	23.0	215	429.590	-0.889	0.375
**zBMI baseline**	0.4	0.9	-3.0	4.2	323	0.5	1.0	-4.0	4.7	215	425.892	-0.884	0.377
**BMI follow-up**	15.8	1.4	12.4	25.8	323	15.8	1.8	11.6	23.1	40	44.957	0.115	0.909
**zBMI follow-up**	0.3	0.9	-2.6	5.4	323	0.2	1.2	-3.3	4.5	39	43.837	0.451	0.654
**CEBQ-FR**	2.0	0.8	1.0	5.0	323	2.1	0.7	1.0	4.3	186	408.605	-0.698	0.486
**CEBQ-EO**	1.5	0.6	1.0	3.7	323	1.6	0.6	1.0	3.5	181	377.536	-1.215	0.225
**CEBQ-EF**	3.5	0.5	1.8	4.6	323	3.6	0.4	2.4	4.4	186	415.172	-1.584	0.114
**CEBQ-SR**	2.9	0.7	1.3	4.8	323	2.9	0.6	1.0	4.3	188	405.854	1.079	0.281
**CEBQ-SE**	2.9	0.8	1.0	5.0	323	2.9	0.7	1.0	4.5	186	399.949	0.056	0.955
**CEBQ-EU**	3.0	0.9	1.0	5.0	323	3.0	0.8	1.0	5.0	184	414.046	0.064	0.949
**CEBQ-FF**	2.8	0.8	1.0	4.8	323	2.9	0.8	1.0	4.8	186	404.156	-0.824	0.410

Mean, SD, Min and ***Max*** refer to the average, standard deviation minimal and maximal value, respectively; ***Val*** refers to valid cases; Descriptives for complete data sets (n=323) are ***appended with .C***, descriptives for incomplete datasets (n = 232 with at least one value missing) are ***appended with .N***; ***Age*** refers to the age in years; ***BMI*** refers to non-adjusted BMI values and ***zBMI*** refers to the age- and sex-adjusted z-transformed BMI at baseline and follow-up (see Methods); ***CEBQ scales*** as follows: ***FR*** refers to Food responsiveness, ***EO*** refers to Emotional overeating, ***EF*** refers to Enjoyment of food, ***SR*** refers to Satiety responsiveness, ***SE*** refers to Slowness in eating, ***EU*** refers to Emotional undereating, and ***FF*** refers to Food fussiness; ***df***, ***statistic*** and ***p-val*** refer to the degrees of freedom, test statistic, and p-value for Welch's test (two-tailed, p-values not corrected for multiple comparisons) with one exception: the frequency of female sex was compared using a Chi-squared test.

Supplementary Table 4: Descriptive statistics of participants with a valid zBMI value at follow-up vs. participants without a valid zBMI at follow-up.

Variable	Mean.C	SD.C	Min.C	Max.C	Val.C	Mean.N	SD.N	Min.N	Max.N	Val.N	df	statistic	p-val
**Age baseline**	3.8	0.7	2.5	6.6	362	3.9	0.7	2.2	6.2	193	354.763	-0.663	0.508
**n Female**	1.5	0.5	1.0	2.0	362	1.5	0.5	1	2	193	390.650	-0.693	0.489
**BMI baseline**	15.9	1.4	12.3	23.0	353	16.1	1.4	11.2	21.2	185	373.785	-1.042	0.298
**zBMI baseline**	0.4	0.9	-3.0	4.7	353	0.5	1.0	-4.0	3.7	185	360.302	-1.061	0.289
**BMI follow-up**	15.8	1.4	11.6	25.8	362	-	-	-	-	-	-	-	-
**zBMI follow-up**	0.3	0.9	-3.3	5.4	362	-	-	-	-	-	-	-	-
**CEBQ-FR**	2.0	0.8	1.0	5.0	341	2.0	0.7	1	4.25	168	351.857	-0.139	0.889
**CEBQ-EO**	1.5	0.6	1.0	3.7	338	1.5	0.5	1	3	166	353.624	-0.395	0.693
**CEBQ-EF**	3.5	0.5	1.8	4.6	339	3.6	0.4	2.4	4.4	170	373.860	-1.923	0.055
**CEBQ-SR**	2.9	0.7	1.3	4.8	341	2.9	0.7	1	4.25	170	341.839	0.686	0.493
**CEBQ-SE**	2.9	0.8	1.0	5.0	339	2.8	0.7	1	4.5	170	347.314	0.725	0.469
**CEBQ-EU**	3.0	0.9	1.0	5.0	339	2.9	0.8	1	5	168	359.764	0.605	0.545
**CEBQ-FF**	2.9	0.8	1.0	4.8	340	2.9	0.8	1	4.8	169	346.451	-0.586	0.558

Mean, SD, Min and ***Max*** refer to the average, standard deviation minimal and maximal value, respectively; Descriptives for complete data sets (n=326 participants with valid zBMI scores at follow up) are ***appended with .C***, descriptives for incomplete datasets (n = 193 without a valid zBMI score at follow-up) are ***appended with .N***; ***Age*** refers to the age in years; ***BMI*** refers to non-adjusted BMI values and ***zBMI*** refers to the age- and sex-adjusted z-transformed BMI at baseline and follow-up (see Methods); ***CEBQ scales*** as follows: ***FR*** refers to Food responsiveness, ***EO*** refers to Emotional overeating, ***EF*** refers to Enjoyment of food, ***SR*** refers to Satiety responsiveness, ***SE*** refers to Slowness in eating, ***EU*** refers to Emotional undereating, and ***FF*** refers to Food fussiness; ***df***, ***statistic*** and ***p-val*** refer to the degrees of freedom, test statistic, and p-value for Welch's test (two-tailed, p-values not corrected for multiple comparisons) with one exception: the frequency of female sex was compared using a Chi-squared test.

Supplementary Table 5: False Discovery Rate (FDR)-Corrected P-Values

Effect	Pooled results after imputation (n= 555)	Only valid cases (n = 323)	Only valid cases, no outliers (n = 303)
**zBMI baseline**	< 0.001	< 0.001	< 0.001
**CEBQ-FR**	0.087	0.157	0.047
**CEBQ-EO**	> 0.999	> 0.999	0.927
**CEBQ-EF**	> 0.999	0.815	0.683
**CEBQ-SR**	> 0.999	0.814	0.165
**CEBQ-SE**	> 0.999	> 0.999	> 0.999
**CEBQ-EU**	0.419	0.428	0.151
**CEBQ-FF**	> 0.999	> 0.999	> 0.999
**SES**	0.009	0.01	0.047

FDR was carried out using the Benjamini-Yekutieli procedure, as implemented in the R "stats" package, for the following nine predictors: ***zBMI***, z-transformed BMI at baseline; ***FR***, Food responsiveness; ***EO***, Emotional overeating; ***EF***, Enjoyment of food; ***SR***, Satiety responsiveness; ***SE***, Slowness in eating; ***EU***, Emotional undereating; ***FF***, Food fussiness; ***SES***, socioeconomic status. Columns 2-4 present FDR-corrected p-values from the pooled model after multiple imputation (see Table 2, main text), the model containing only valid cases (Supplementary Table 1), and the model containing only valid cases and no outliers (Supplementary Table 2), correspondingly.

3 Supplementary Figures

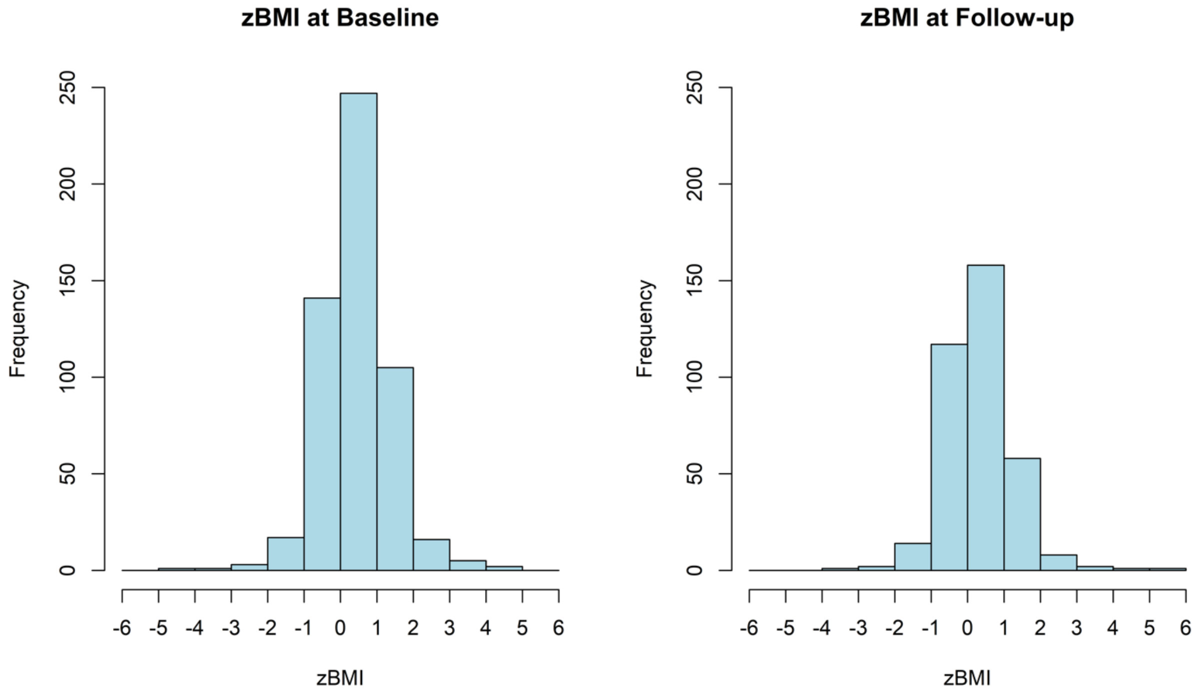

Supplementary Figure 1

Left, distribution of zBMI-values at baseline; ***Right***, distribution of zBMI-values at follow-up (1. Wave, n = 538; 2. Wave, n = 362). Histogram bars represent intervals that are left-open and right-closed: the first bar represents values in the range (-6, -5], i.e., larger than -6 and equal to or smaller than -5, the second – only values in the range (-5, -4], i.e. larger than -5 and equal to or smaller than -4, and so on.

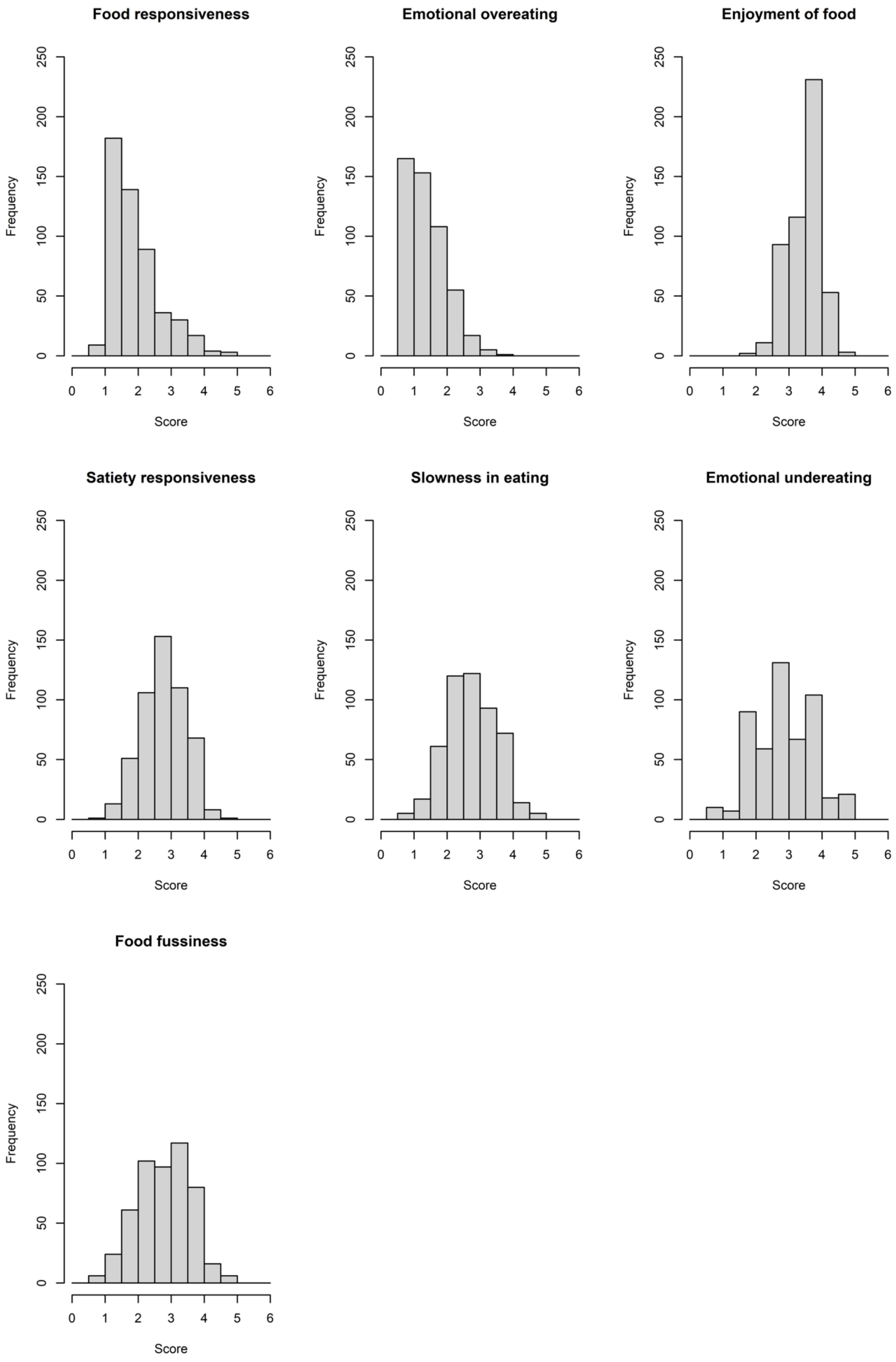

Supplementary Figure 2.

Distribution of CEBQ Scale Scores at Baseline. Valid cases as follows: Food responsiveness, n = 509; Emotional overeating, n = 504; Enjoyment of food, n = 509; Satiety Responsiveness, n = 511; Slowness in eating, n = 509; Emotional undereating, n = 507; Food fussiness, n = 509. Histogram bars represent intervals that are left-open and right-closed: the first bar represents values in the range (0, 0.5], i.e., larger than 0 and smaller than or equal to 0.5, the second – in the range (0.5, 1], i.e., values larger than 0.5 up to and including 1.0, and so on.

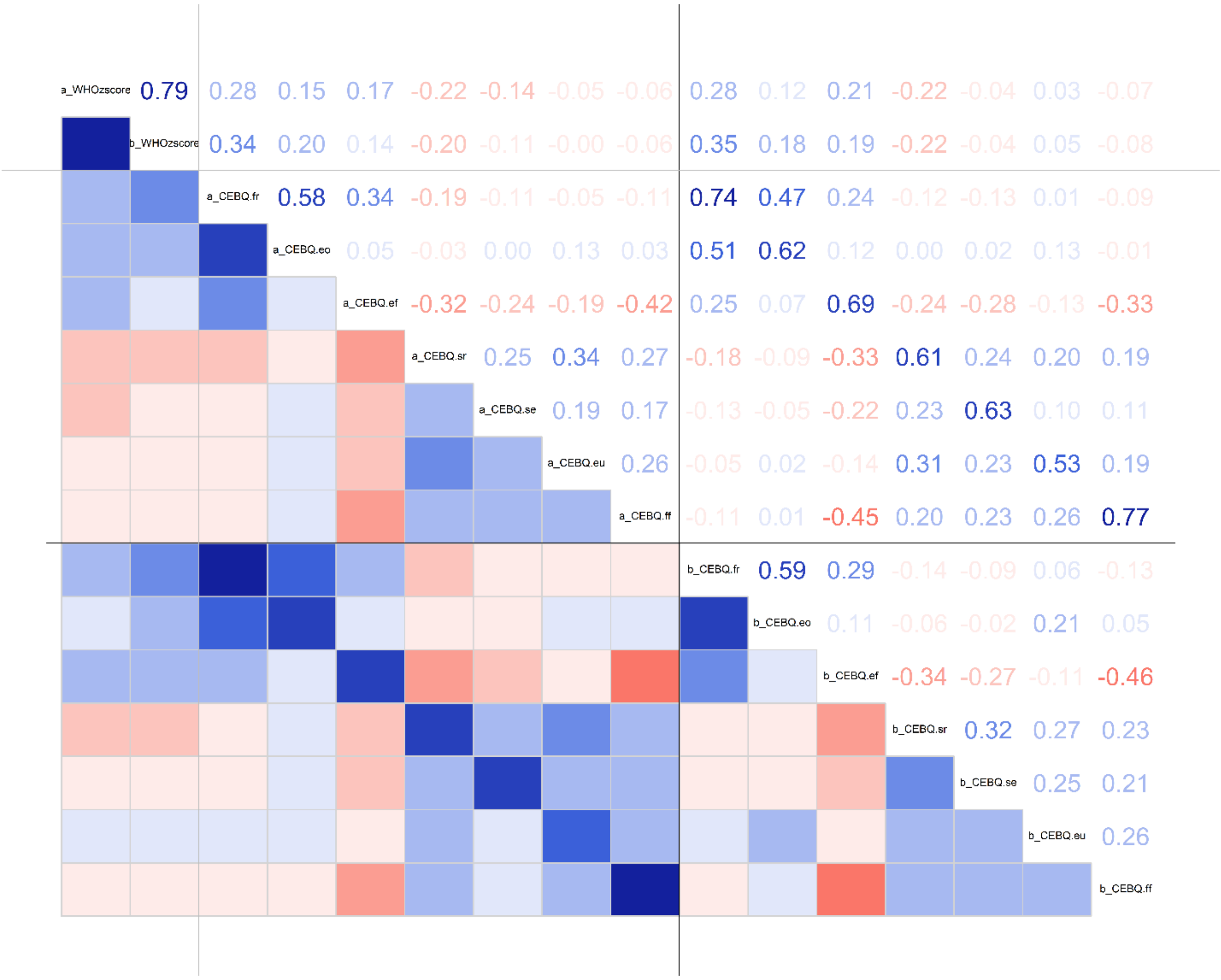

Supplementary Figure 3

A correlogram, summarizing the pattern of correlations between predictors and outcome. Variables are listed in the ***diagonal***, with a ***prepended a_*** referring to baseline and a ***prepended b_*** referring to follow-up. ***WHOzscores*** refer to z-standardized BMI, ***CEBQ*** refers to CEBQ scales abbreviated as in Supplementary Tables 1-4. ***Above the diagonal***, Pearson's r correlation coefficients based on the sample with n = 555. ***Below the diagonal***, a color code for correlations. To enhance readability, a ***gray cross in the upper left corner*** separates z-standardized BMI scores from CEBQ scales. Likewise, to enhance readability, a ***black cross in the middle*** of the image separates between CEBQ scales from the first and second wave.

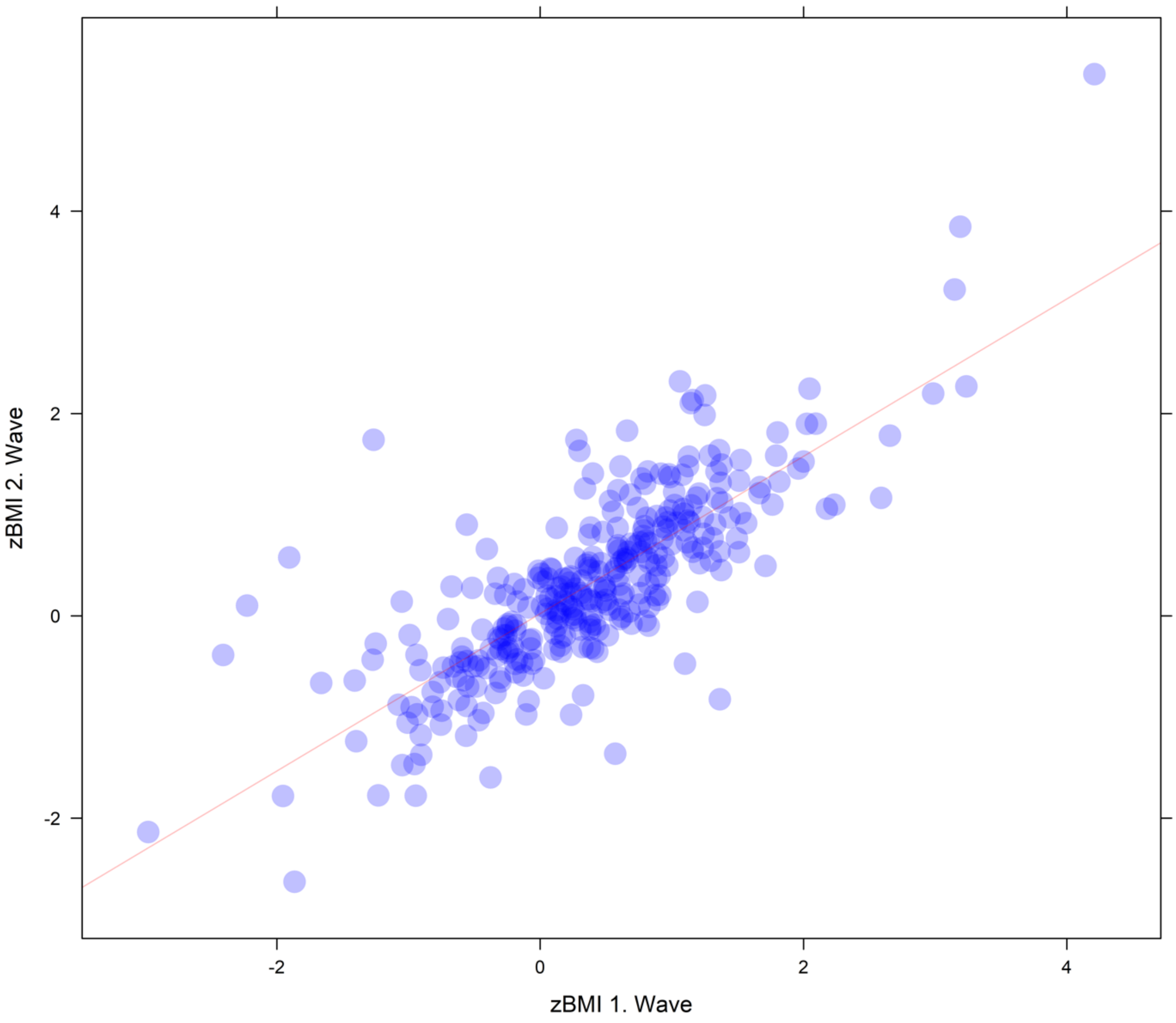

Supplementary Figure 4

Semitransparent dots represent individual participant data. Due to semi-transparency, dot color incrementally changes from light blue to dark blue when participants overlapped, i.e., when more than one participant showed the same combination of values. The ***red line*** represents a linear regression estimate, as generated by the function “xyplot” in the R package “lattice” (version 0.20.45). ***zBMI*** refers to age- and sex-adjusted z-transformed BMI; ***1. Wave*** and ***2. Wave*** refer to baseline and one-year follow-up, correspondingly. Valid cases, ***n = 323***.

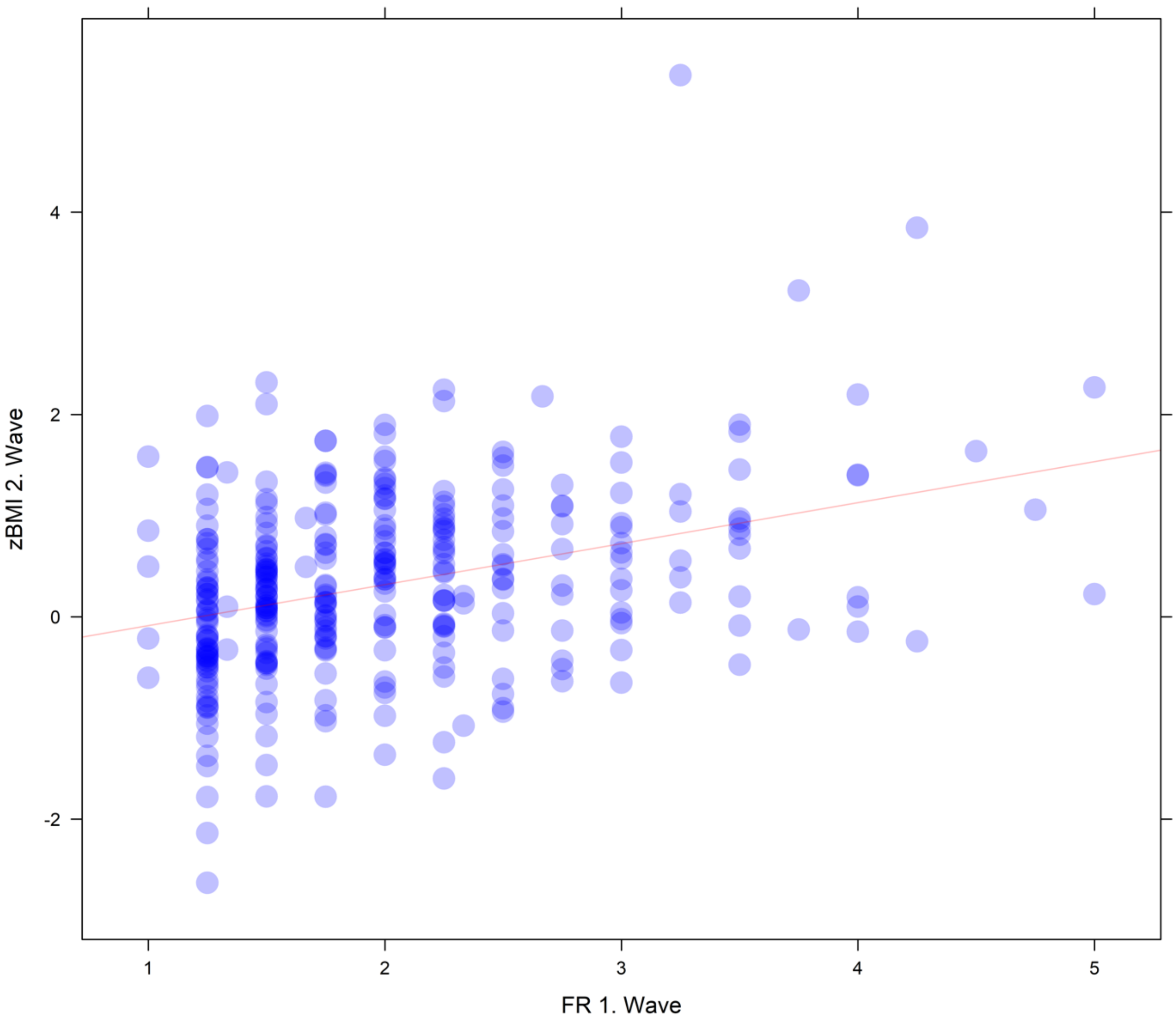

Supplementary Figure 5

Semitransparent dots represent individual participant data. Due to semi-transparency, dot color incrementally changes from light blue to dark blue when participants overlapped, i.e., when more than one participant showed the same combination of values. The ***red line*** represents a linear regression estimate, as generated by the function “xyplot” in the R package “lattice” (version 0.20.45). ***zBMI*** refers to age- and sex-adjusted z-transformed BMI; ***1. Wave*** and ***2. Wave*** refer to baseline and one-year follow-up, correspondingly; ***FR*** refers to food responsiveness scores. Valid cases, ***n = 323***.
